# *QuickStats:* Age-Adjusted Death Rates* from Stroke^†^ Among Adults Aged ≥65 Years, by Race and Hispanic Origin — National Vital Statistics System, United States, 2000–2020

**DOI:** 10.15585/mmwr.mm7141a5

**Published:** 2022-10-14

**Figure:**
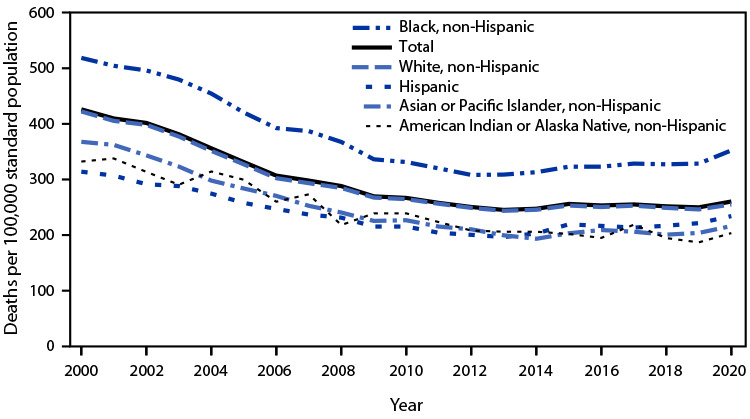
Age-adjusted death rates from stroke among adults aged ≥65 years generally declined from 425.9 deaths per 100,000 standard population in 2000 to 250.0 in 2019 before increasing to 260.5 in 2020. During 2019–2020, stroke death rates increased for Hispanic adults (from 221.6 to 234.0), non-Hispanic Asian or Pacific Islander adults (from 203.9 to 216.4), non-Hispanic Black adults (from 328.4 to 352.2), and non-Hispanic White adults (from 246.2 to 255.0); changes for non-Hispanic American Indian or Alaska Native adults were not significant. Throughout the 2000–2020 period, death rates for non-Hispanic Black adults were higher than those for adults in other race and Hispanic origin groups.

**Source:** National Vital Statistics System, Mortality Data. https://www.cdc.gov/nchs/nvss/deaths.htm

